# Transient Postoperative Myoclonus Following Hypothermic Circulatory Arrest

**DOI:** 10.31486/toj.25.0085

**Published:** 2026

**Authors:** Brock Lingle, Taylor Lambert, Sydney Claypoole, Harsahib Dev, Hunter Douglas, Bobby D. Nossaman

**Affiliations:** ^1^ Department of Anesthesiology, Tulane University School of Medicine, New Orleans, LA; ^2^ Department of Anesthesiology and Perioperative Medicine, Ochsner Clinic Foundation, New Orleans, LA; ^3^ Department of Surgery, Ochsner Clinic Foundation, New Orleans, LA; ^4^ The University of Queensland Medical School, Ochsner Clinical School, New Orleans, LA

**Keywords:** *Aorta*, *apraxias*, *circulatory arrest–deep hypothermia induced*, *complications*, *myoclonus*, *neurologic manifestations*, *surgery*

## Abstract

**Background:**

Neurologic complications are a risk of aortic arch surgery performed with hypothermic circulatory arrest. Stroke, encephalopathy, and seizures are well-recognized complications, whereas postoperative movement disorders are rarely reported.

**Case Report:**

A 72-year-old male underwent elective ascending aorta and hemiarch replacement under hypothermic circulatory arrest (28 minutes, nadir 28.9 °C) with bilateral antegrade cerebral perfusion and stable cerebral oximetry readings. During postoperative weaning from sedation, the patient developed persistent bilateral myoclonus, diffuse rigidity, and suspected eyelid apraxia, present at rest and unaffected by voluntary movement. Although a stroke code was activated, computed tomographic angiography of the head and neck was normal. Laboratory evaluation showed mild hypocalcemia without other metabolic derangements. Electroencephalography and magnetic resonance imaging were not performed. The patient was treated with intravenous levetiracetam and calcium supplementation. The movements resolved by postoperative day 3, and the patient was discharged home neurologically intact on day 5.

**Conclusion:**

This case demonstrates a transient form of postoperative myoclonus distinct from Lance-Adams syndrome. Recognition of atypical movement disorders after hypothermic circulatory arrest may help clinicians avoid unnecessary escalation of therapy and provide reassurance regarding the potential for complete recovery.

## INTRODUCTION

Hypothermic circulatory arrest allows safer repair of complex aortic pathology but carries the risk of neurologic complications. Contemporary studies still report neurologic complications, including stroke and transient neurologic dysfunction, in up to 30% of patients despite adjunctive perfusion strategies.^1,2^ Movement disorders are rarely described.

Perioperative myoclonus is often attributed to posthypoxic conditions such as Lance-Adams syndrome, a condition that typically emerges days after hypoxic insult, is action-sensitive, and follows a chronic course.^3^ We describe an unusual case of immediate postoperative myoclonus following hypothermic circulatory arrest that rapidly and completely resolved.

## CASE REPORT

A 72-year-old male with hypertension, hyperlipidemia, coronary artery disease (on aspirin), and hypothyroidism was scheduled for elective ascending aorta and hemiarch replacement for an enlarging 5.1-cm aneurysm. The patient had no prior neurologic events, relevant psychosocial issues, relevant surgical interventions, or contributory family history. Preoperative echocardiography showed a preserved left ventricular ejection fraction of 55% to 60%.

Cardiopulmonary bypass was initiated with right atrial and ascending aortic cannulations. Selective bilateral antegrade cerebral perfusion and topical cooling were used. Circulatory arrest lasted 28 minutes, the aortic cross-clamp time was 82 minutes, and nadir temperature was 28.9 °C. Regional cerebral oxygen saturation remained stable (>60%) throughout the cardiopulmonary bypass period.

During initial weaning of sedation in the intensive care unit, the patient exhibited persistent bilateral upper extremity myoclonus (greater in arms than legs), generalized rigidity, and eyelid apraxia. A stroke code was activated. Computed tomographic angiography of the head and neck demonstrated no acute infarct, hemorrhage, or large vessel occlusion. Neurology concluded that the findings were not consistent with a cerebrovascular event.

Neurologic examination showed an alert, oriented patient without cranial nerve deficits. Movements were constant at rest and unaffected by voluntary activity. Laboratory results included anemia (hemoglobin 10.0 g/dL; reference range, 13.5-15.0 g/dL) and mild hypocalcemia (7.6 mg/dL; reference range, 8.5-10.0 mg/dL, corrected). No other electrolyte or metabolic abnormalities were noted.

Initially, the observed myoclonus following orotracheal extubation was interpreted as postoperative shivering. Intravenous meperidine (25 mg) was administered, which had no effect. Following neurologic consultation, the patient was treated with intravenous levetiracetam (2 g loading dose and 1 g every 12 hours for 1 day), followed by 500 mg orally twice daily throughout the patient's remaining hospital stay.

Movements gradually improved and resolved by postoperative day 3. Eyelid apraxia and weakness resolved by day 5. The patient tolerated levetiracetam without adverse effects. The patient was discharged home for self-care neurologically intact and remained asymptomatic at 3-week follow-up.

## DISCUSSION

Neurologic morbidity remains a defining concern of aortic arch surgery performed with hypothermic circulatory arrest. Ergin et al reported that temporary neurologic dysfunction occurred in approximately 28% of evaluated patients, with longer durations of circulatory arrest identified as a significant risk factor.^2^ Our patient's arrest time was 28 minutes with bilateral antegrade cerebral perfusion and stable cerebral oximetry. However, stable cerebral oximetry may not detect focal or transient cortical ischemia and thus cannot fully exclude subclinical neurologic injury.

Perioperative myoclonus is often attributed to Lance-Adams syndrome, which is characterized by action-induced jerks that develop days after hypoxic injury in conscious patients.^3^ Myoclonic status epilepticus, by contrast, occurs in comatose patients after hypoxic-ischemic injury and is associated with poor neurologic prognosis.^4^ Our case diverged from both patterns: neurologic onset occurred during initial weaning from sedation, movements were present at rest, and symptoms rapidly resolved.

Possible contributors to this patient's myoclonus include transient cortical hyperexcitability related to reperfusion, subclinical ischemia despite preserved oximetry, and metabolic vulnerability from mild hypocalcemia. Although electroencephalography and magnetic resonance imaging were not performed, the benign clinical course argues against significant structural injury.

For anesthesiologists and intensivists, awareness of this atypical postoperative syndrome is important. Misdiagnosis as stroke, myoclonic status epilepticus, or Lance-Adams syndrome may prompt unnecessary escalation, whereas exclusion of structural injury, correction of reversible contributors, and close follow-up may suffice in selected cases.

## CONCLUSION

We describe a transient postoperative myoclonus distinct from Lance-Adams syndrome occurring after hypothermic circulatory arrest with bilateral antegrade cerebral perfusion for aortic arch surgery. The patient's rapid and complete recovery highlights the potential for benign, reversible movement disorders in this setting. Recognition of this phenomenon may prevent misdiagnosis, avoid unnecessary interventions, and provide reassurance to patients and families.

## Notes

*This article meets the Accreditation Council for Graduate Medical Education and the American Board of Medical Specialties Maintenance of Certification competencies for Patient Care and Medical Knowledge.*
